# Sustainable production of glutaric acid in microbial cell factories: Current advances and future prospects

**DOI:** 10.1016/j.synbio.2026.01.003

**Published:** 2026-01-23

**Authors:** Jie Liu, Dan Mei, Xuan-Jun Zhang, Wei-Guo Zhang, Long-Bao Zhu

**Affiliations:** a School of Biological and Food Engineering, Anhui Polytechnic University, 18^#^Beijing Middle Road, WuHu, 241000, People's Republic of China; b The Key Laboratory of Industrial Biotechnology, Ministry of Education, School of Biotechnology, Jiangnan University, 1800^#^ Lihu Road, WuXi, 214122, People's Republic of China

**Keywords:** Glutaric acid, Green manufacturing, Microbial cell factories, Sustainable production, Metabolic engineering

## Abstract

Glutaric acid is a significant C5 dicarboxylic acid, extensively utilized in the chemical industry, medicine, and biomaterials. In recent years, the advancement of synthetic biology and metabolic engineering has rendered microbial production of glutaric acid a sustainable alternative to conventional chemical synthesis. This study reviews recent advancements in glutaric acid biosynthesis, primarily concentrating on the design of biosynthetic pathways and metabolic engineering strategies for the development of engineered strains. The utilization of systems biology technologies in the development of the glutaric acid biosynthetic pathway is examined. This study outlines the issues associated with glutaric acid biosynthesis and its prospective developmental trajectory, intending to offer theoretical insights and technological guidance for the sustainable production of glutaric acid and related fine chemicals.

## Graphical abstract

**Figure:**
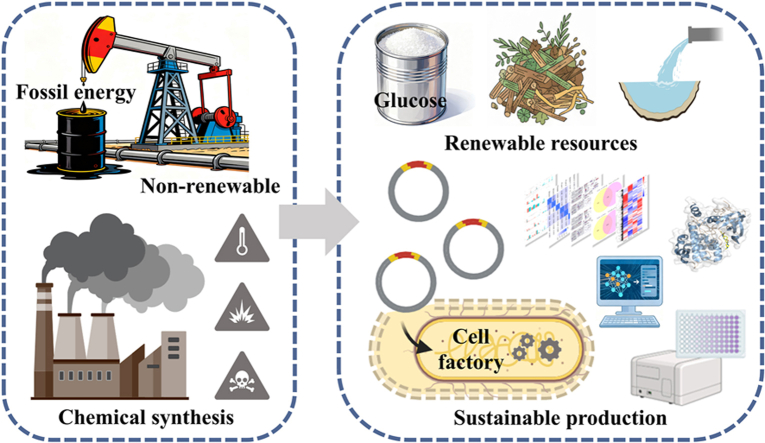

## 1 Introduction

The microbial cell factory, as an innovative biological manufacturing platform, achieves precise regulation of the biosynthesis pathway for target products through systematic metabolic network design and directed transformation, thereby efficiently synthesizing high value-added chemicals and novel functional compounds [[1], [2], [3]]. With the escalation of global climate change and the depletion of fossil resources, the establishment of efficient microbial cell factories utilizing renewable feedstocks has emerged as a pivotal strategy to supplant conventional petrochemical production [[4], [5], [6], [7], [8]]. Glutaric acid, a significant C5 platform compound, serves as the primary raw material for the synthesis of polyamide, polyurethane, glutaric anhydride, and other high-performance materials, in addition to being a crucial chemical intermediate for 1,5-pentanediol and 5-hydroxyvaleric acid [9,10]. Nonetheless, the conventional chemical synthesis process typically exhibits significant drawbacks, including reliance on non-renewable raw materials, intricate reaction sequences, elevated energy consumption for product separation, and substantial environmental impact [11]. In this context, the development of an efficient glutaric acid biosynthesis cell factory can substantially diminish reliance on fossil resources while fostering a green, low-carbon, and sustainable industrial chain, thereby possessing significant economic value and ecological importance.

Despite the gradual achievement of glutaric acid production from l-lysine via whole-cell catalysis in a buffer-free one-pot reaction, and the development of green synthesis strategies utilizing hydrogen peroxide and tungstic acid, these methods presently face challenges in serving as viable alternatives to chemical synthesis due to suboptimal product yields and elevated production costs [[12], [13], [14]]. Conversely, microbial fermentation exhibits superior industrialization potential owing to its distinct advantages: microorganisms can exploit inexpensive renewable carbon sources like lignocellulose hydrolysate, and fermentation technology is characterized by ease of scale-up, while metabolic engineering can markedly enhance strain performance [[15], [16], [17]]. Given that glutaric acid is a natural product synthesized by *Pseudomonas putida* from l-lysine, metabolic engineers effectively developed glutaric acid biosynthetic engineering bacteria by integrating the lysine degradation pathway of *P. putida* into industrial strains that produce l-lysine (*Escherichia coli* and *Corynebacterium glutamicum*) [9,18]. The current development of glutaric acid biosynthetic strains primarily employs metabolic engineering techniques, including gene overexpression, knockout, and transcriptional regulation (such as promoter replacement and codon optimization), with the objective of systematically manipulating the microbial metabolic network to enhance the final yield of glutaric acid and the substrate conversion rate. In recent years, systems metabolic engineering, an advanced phase of metabolic engineering, empowers researchers to meticulously design and construct microbial cell factories with efficient target product synthesis capabilities on a systemic scale by integrating multidisciplinary, cutting-edge technologies such as systems biology, synthetic biology, computational biology, enzyme engineering, and artificial intelligence [4,19]. Furthermore, an in-depth examination of metabolic control mechanisms and their application in the rational design of engineered strains offers theoretical guidance for the metabolic network remodeling of microbial cell factories [20,21]. The utilization of sophisticated technology to develop an effective microbial cell factory for glutaric acid production represents a significant advancement in achieving sustainable biological manufacturing.

Glutaric acid, a significant C5 platform compound, holds considerable strategic importance in advancing green manufacturing technology innovation, achieving sustainable chemical production, and facilitating industrial low-carbon transition. This study carefully reviews the most recent advancements in this sector. Initially, the production pathways of glutaric acid, both natural and artificially engineered, together with their metabolic regulatory systems, are thoroughly examined. The construction methodologies for glutaric acid biosynthesis cell factories are summarized, with a particular emphasis on the application of systems metabolic engineering. The potential application of artificial intelligence-assisted design and other advanced technologies in the efficient synthesis of glutaric acid is anticipated, with the objective of offering practical direction for future study.

## 2 Natural and engineered pathways for glutaric acid biosynthesis

To date, glutaric acid can be biosynthesized through four metabolic pathways, including lysine catabolism [22,23], α-ketoglutarate (α-KG) reduction [24], carbon chain extension and decarboxylation of α-KG [25] and reverse β-oxidation [11].

**Fig. 1:**
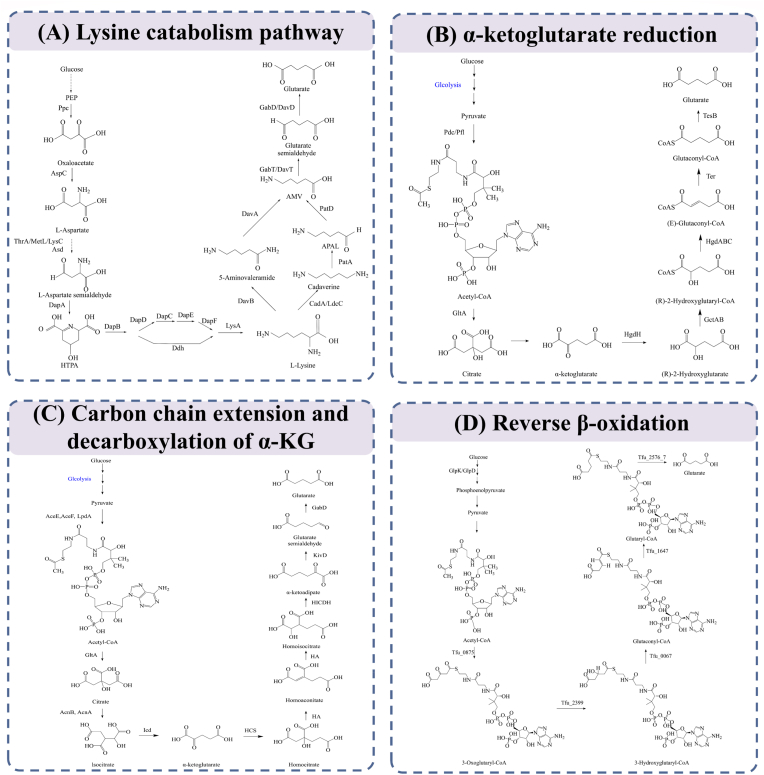
Four biosynthetic pathways for glutaric acid production from glucose.

Lysine can be converted to glutaric acid by two different catabolic pathways during microbial metabolism: 5-aminopentanoate (AMV) pathway and cadaverine pathway (Fig. 1A) [26,27]. The AMV pathway, which occurs naturally in *P. putida*, is a four-step enzymatic process: the initial reaction is catalyzed by lysine 2-monooxygenase (DavB), followed by delta-aminopentamidase (DavA), 5-aminopentanoate aminotransferase (DavT) and glutarate semialdehyde dehydrogenase (DavD) to produce glutaric acid [28]. *C. glutamicum*, a significant lysine-producing strain in industry, contains endogenous 5-aminopentanoate aminotransferase (GabT) and glutarate semialdehyde dehydrogenase (GabD), rendering it an optimal host for the development of a glutaric acid biosynthetic system [29]. In contrast to *C. lutamicum*, *E. coli* utilizes the endogenous cadaverine pathway to transform lysine into glutarate, encompassing five critical enzymatic reactions: lysine is decarboxylated to cadaverine by lysine decarboxylase (CadA or LdcC); cadaverine aminotransferase (PatA) facilitates its transamination to 5-aminopentanal, which is subsequently oxidized to glutarate semialdehyde by aminopentanal dehydrogenase (PatD); finally, GabT and GabD collaborate to convert it to glutarate [30]. Despite the cadaverine pathway involving an additional reaction step compared to the AMV pathway, its anaerobic metabolic properties confer distinct advantages in industrial fermentation, including suitability for large-scale production under hypoxic or anaerobic conditions, which subsequently diminishes energy consumption and enhances economic viability [31].

Researchers have developed an innovative α-ketoglutarate reduction pathway in recombinant *E. coli*, utilizing α-ketoglutarate as the primary substrate to facilitate glutarate production via multi-step enzyme-catalyzed reactions [24]. Specifically, Alpha-ketoglutarate is initially reduced to (R)-2-hydroxyglutarate, catalyzed by 2-hydroxyglutarate dehydrogenase (*hgdH*) sourced from *Acidaminococcus fermentans* DSM 20731. Subsequently, (R)-2-hydroxyglutarate is activated to its CoA thioester form by glutaconate CoA-transferase (*gctAB*) from the same strain. The activation product was subsequently produced in 2-hydroxyglutaryl-CoA dehydratase (*hgdAB*) from *Clostridium symbiosum* and its activating protein *hgdC* from *A. fermentans* DSM 20731, resulting in the dehydration of (E)-pentenoyl-CoA. In a later step, (E)-pentenoyl-CoA is reduced to glutaryl-CoA, mediated by the mutant *trans*-enoyl-CoA reductase (*terI* 287V) derived from *Treponema denticola* ATCC 35405. Ultimately, thioesterase (*tesB*) from *E. coli* MG1655 catalyzes the hydrolysis of glutaryl-CoA, resulting in the liberation of free glutarate (Fig. 1B).

A separate study established an innovative *E. coli*-derived biosynthetic pathway for glutaric acid, incorporating two essential metabolic modules: “carbon chain elongation” and “α-ketoacid decarboxylation” [25]. Metabolic engineers initially developed the carbon chain extension pathway for α-ketoglutarate (the “+1” extension mechanism) through the heterologous expression of an engineered enzyme system from the tricarboxylic acid cycle of *Saccharomyces cerevisiae*, comprising homocitrate synthase (HCS), homaconitase (HA), and homocitrate dehydrogenase (DHDH) (Fig. 1C). The transformation of α-ketoadipic acid into glutaric acid was accomplished through the overexpression of α-ketoacid decarboxylase (encoded by *kivD*) and succinate semialdehyde dehydrogenase (encoded by *gabD*), both of which are native to *E. coli*. The metabolic characterisation of this pathway reveals that the synthesis of one molecule of glutaric acid results in the production of two CO_2_ molecules, attributed to carbon chain elongation and decarboxylation activities, respectively.

The reverse β-oxidation pathway is predicated on six principal enzyme-coding genes of *Bifidobacterium griseus*: *tfu_0875* (encoding β-ketothiolase), *tfu2399* (encoding 3-hydroxyacyl-CoA dehydrogenase), *tfu0067* (encoding 3-hydroxyadipyl-CoA dehydrogenase), *tfu1647* (encoding 5-carboxy-2-pentenoyl-CoA reductase) and *tfu2576-7* (encoding adipyl-CoA synthetase) (Fig. 1D). This process can biosynthesize glutarate utilizing malonyl coenzyme A and acetyl coenzyme A as precursors, resembling the α-ketoacid reduction pathway without carbon dioxide generation [11].

The development of the α-ketoglutarate (α-KG) reduction pathway fills a gap in the synthesis of glutaric acid from *trans*-glutaryl coenzyme A and is suitable for glutaric acid production in *E. coli* hosts. The carbon chain extension-decarboxylation pathway of α-KG achieves glutaric acid synthesis through α-ketoacid-mediated carbon chain extension and oxidative decarboxylation in *E. coli*, which helps researchers avoid cumbersome steps in the construction of lysine-producing strains or in the screening for highly active enoate reductases, and involves fewer enzymatic reaction steps. The reverse β-oxidation pathway has advantages in solving the problems of limited lysine supply and long fermentation cycle. This pathway does not require the addition of lysine and has a shorter fermentation time, which significantly improves the feasibility of bio-based glutaric acid production. Among the four reported mechanisms of glutaric acid biosynthesis, the lysine catabolic pathway is considered the most promising due to its elevated theoretical yield and practical applicability [9]. Metabolic flux research indicates that the theoretical molar production of this system can attain 0.75 mol/mol glucose, markedly surpassing the alternative pathway reliant on α-ketoglutarate, which yields 0.67 mol/mol glucose [25,32]. Furthermore, by the optimization of metabolic engineering techniques, including the augmentation of critical enzyme expression and the minimization of by-product accumulation, lysine-derived glutaric acid synthesis has attained elevated titers above 100 g/L at the laboratory scale, indicating significant commercial potential.

## 3 Metabolic engineering strategy for glutaric acid biosynthetic strains construction

For *P. putida* KT2440, which naturally has a glutaric acid anabolic pathway, Zhang et al. first blocked two glutaric acid catabolic pathways in the recombinant strain by deleting glutaryl-CoA dehydrogenase (encoded by *gdh*) and Fe^2+^/2-KG-dependent glutarate hydroxylase (encoded by *cisD*) [33]. The alanine racemase-encoding gene *alr* was then removed to inhibit the conversion of the primary precursor, l-lysine, to d-lysine. The modified strain KT2440 (Δ*gdh*Δ*csiD*Δ*alr*) produced 1.94 g/L of glutaric acid in a medium with 5 g/L glucose and 5 g/L l-lysine, yielding 0.85 mol glutaric acid/mol. However, the production of glutaric acid by *P*. *putida* still faces many limitations. For example, the fermentation process of *P. putida* necessitates rigorous control over dissolved oxygen and pH, particularly the exceptionally high demand for oxygen, which raises the control cost of large-scale production [34,35]. Therefore, researchers choose to use model industrial microbes *E.* *coli* or *C. glutamicum* as alternate hosts for glutaric acid production due to their clear genetic background, rapid growth rate, high metabolic adaptability, and ease of scale-up production [30,32]. Rapid advances in biotechnology have led to the establishment and optimization of glutaric acid biosynthetic pathways in *E*. *coli* and *C*. *glutamicum* based on metabolic engineering and systems metabolic engineering (Table 1).

**Table 1:** Production performance of glutaric acid synthesis strains constructed by metabolic engineering.

Strain	Characteristic	Titer (g/L)	Productivity	Reference
***E. coli***				
**WL3110**	Overexpression of *gabTD* and *davAB* genes from *P. putida*	1.7	0.085 g/g	[36]
**BW25113(DE3)** **DcadA DldcC pCDF-lysC^fbr^-dapA^fbr^-pSTV-*davBA*-pTrc*davDT***	Introduction of *dapA*^fbr^ and *lysC*^fbr^, deletion of *cadA* and *ldcC*, overexpression of *davAB* and *davDT*	0.82	68 mmol/mol	[30]
**BM31PER**	Expression of *cadA* via low copy plasmid, *patAD* and *gabTD* via medium copy plasmid, overexpression of AMV transporter GabP and cadaverine transporter PotE, deletion of transcription factor IclR that inhibits glyoxylate bypass operator expression	54.5	0.54 mol/mol	[41]
**Bgl1468**	Expression of *matB* (malonic acid synthase) and *matC* (malonic acid carrier protein) from *Clover rhizobia* increases malonic acid supply.	6.3	–	[111]
**Glu-02**	Ribosome binding site regulation was used to coordinate the enzyme molar ratio EcCA: KpcPA: KpcPD to about 4:8:10 and overexpression of the 5AVA transporter GabP.	77.62	0.78 g/g	[43]
**RY29**	Biosensor-based optimization of gene copy number of key enzymes in glutaric acid synthesis pathway, overexpression of glutaric acid transporter YidE and lysine transporter LysP.	44.8	0.28 g/g	[42]
***C. glutamicum***				
**GRLys1Δ*sugR*Δ*ldhA*Δ*snaA*Δ*cgmA*Δ*gdh*(pVWEx1-*ldcC*)(pEKEx3-*patDA*)(pECXT99A-*gabTD*^Stu^)**	*ldcC*, *patA* and *patD* from *E. coli* and *gabTD*^Pstu^ from *Pseudomonas stutzeri* ATCC 17588 were overexpressed and the genes *sugR*, *ldhA*, *snaA*, *cgmA* and *gdh* were knocked out.	25	0.17 g/g	[23]
**GTA-4**	*gabTD* is regulated using the *tuf* promoter and the 5-aminopentanoate transporter NCgl0464 is overexpressed.	90	0.70 mol/mol	[32]
**H30_GAHis**	Expression of codon-optimized *davTDBA* from *P. putida* and introduction of His 6-tag in N-terminal region of *davT* and *davB*	24.5	–	[22]
**GA17 (pGA4)**	Overexpressing *davBA* from *P. putida* and *gabTD* from *C. glutamicum*, adjusting expression levels of 11 target genes based on genomic analysis, and overexpressing glutaric acid export protein-encoding gene *ynfM*.	105.3	0.54 g/g	[29]
**GTA-3**	Increase the expression level of *davBA* gene and introduce NADH-dependent enzyme to replace NADPH-dependent enzyme in l-lysine biosynthesis pathway.	65.6	–	[40]

### 3.1 Conventional metabolic engineering

Conventional metabolic engineering seeks to develop and enhance the glutarate production pathway by overexpressing important enzyme genes, tweaking promoters/regulatory elements, knocking off competitor routes, and adding foreign genes.

Recent years have seen the development of various biosynthetic routes aimed at the sustainable production of glutaric acid in *E. coli*. Park et al. heterologously expressed *davAB* and *gabTD* gene clusters from *P*. *putida*, resulting in recombinant *E. coli* WL3110 producing 1.7 g/L glutaric acid in a medium containing 20 g/L glucose, 10 g/L l-lysine and 10 g/L α-ketoglutarate (α-KG) [36]. In a concurrent study, Adkins et al. restricted the formation of the byproduct cadaverine to increase the accumulation of l-lysine, a precursor to glutaric acid synthesis, by introducing feedback resistant mutants of aspartate kinase III and dihydropicolinate synthase in *E. coli* BW25113(DE3) and further deleting the genes *cadA* and *ldcC* encoding the native lysine decarboxylase [30]. The glutaric acid biosynthetic pathway of *P*. *putida* was introduced on this basis, and the obtained engineering strain produced only 0.82 g/L glutaric acid from glucose. This signifies that the efficiency of glutarate production in *E. coli* is markedly constrained by the availability of α-KG for the critical enzyme aminopentanoic acid aminotransferase. In order to avoid the burden of α-KG addition on production costs, Wang et al. designed a new alternative glutaric acid biosynthesis pathway, namely the α-KG carbon chain extension pathway described above [25]. CRISPRi inhibits *sucA* and *sucB* in the TCA cycle, leading to an increased reintroduction of α-KG into the carbon chain elongation pathway, culminating in a final strain that produces 0.42 g/L of glutarate. In addition, Zhao et al. implemented a five-step reverse beta-oxidation pathway derived from *Bifidobacterium fusiforme* into *E. coli* [11]. The findings indicated that the modified *E. coli* strains produced a minimal quantity of glutaric acid. To enhance the product titer, the optimization of the culture medium, culture mode, inducer, and inhibitor was initially conducted. Induction was performed using 0.8 mM IPTG, and the blockage of the fatty acid synthesis pathway was achieved with 0.18 mM cerulenin as an antibiotic under microaerobic conditions in the optimum SOB medium. The CRISPR/Cas9 system eliminates essential enzyme genes that compete for metabolites like lactic acid, butyric acid, and formic acid to enhance metabolic flux. In fed-batch fermentation with glycerol as the primary substrate, the glutaric acid concentration of the final engineered strain Bgl 4146 attained 36.5 mM within 80 h. Despite numerous investigations aiming to develop glutamate-dependent glutaric acid synthesis pathways in *E. coli,* traditional metabolic engineering approaches have proven ineffective for efficient production.

*C. glutamicum* has been effectively modified to synthesize various C5 platform chemicals, including gamma-aminobutyric acid (GABA), cadaverine, 1,5-pentanediol (1,5-PDO), and 5-aminovaleric acid (5AVA) [8,[36], [37], [38], [39]]. *C. glutamicum* is regarded as the optimal host for industrial glutaric acid production, capable of large-scale l-lysine synthesis utilizing cost-effective substrates [26,31]. In contrast to *E. coli* and *P. putida*, *C. glutamicum* does not possess an l-lysine degradation pathway. The glutaric acid production in *C*. *glutamicum* can be achieved by incorporating the essential enzyme gene cluster *davTDBA* from the glutaric acid biosynthesis pathway of *P. putida*. The expression of codon-optimized *davTDA* and His-tagged *davB* in recombinant *C. glutamicum* KCTC 1857, regulated by a robust synthetic H30 promoter, established a glutaric acid biosynthetic pathway. The altered *C. glutamicum* H30_GAHis yielded 24.5 g/L of glutaric acid and accumulated 1.7 g/L of l-lysine during fed-batch fermentation [22]. Pérez-García et al. metabolically engineered l-lysine producing strains to construct engineered glutaric acid producing strains by introducing lysine decarboxylase, putrescine aminotransferase and g-aminobutyraldehyde dehydrogenase from *E*. *coli*, and GABA/5AVA aminotransferase and succinate/glutarate semialdehyde dehydrogenase from *Clostridium glutamicum* or three *P*. *putida* species [23]. Deletion of acetylase and export genes was implemented to prevent carbon loss associated with the production of byproducts cadaverine and N-acetyl cadaverine. Furthermore, *gdh* was deleted to integrate glutaric acid overproduction with l-glutamate biosynthesis, which encodes glutamate dehydrogenase. Consequently, the resulting strain necessitated a transamination reaction for glutaric acid overproduction to generate l-glutamate, thereby compensating for the deficiency in glutamate dehydrogenase. The final strain produces glutaric acid at a concentration of 25 g/L through fed-batch fermentation, exhibiting a volumetric productivity of 0.32 g/L/h. Sohn et al. constructed two transformation modules based on two-vector system, namely l-lysine transformation module and glutaric acid production module, to carry out metabolic engineering on *C. glutamicum* [40]. The production of l-lysine was augmented by substituting NADPH-dependent enzymes with NADH-dependent enzymes in the l-lysine biosynthetic pathway. Subsequently, the optimization of the glutaric acid biosynthesis pathway was achieved by enhancing the expression levels of the *davB* and *davA* genes, concurrently minimizing byproduct accumulation. Engineered strains expressing *dapB* mutants from *E. coli* (*dapBC*^115G, G116C^) produced 65.6 g/L glutaric acid in 5 L fed-batch fermentation experiments.

### 3.2 Systems metabolic engineering

**Fig. 2:**
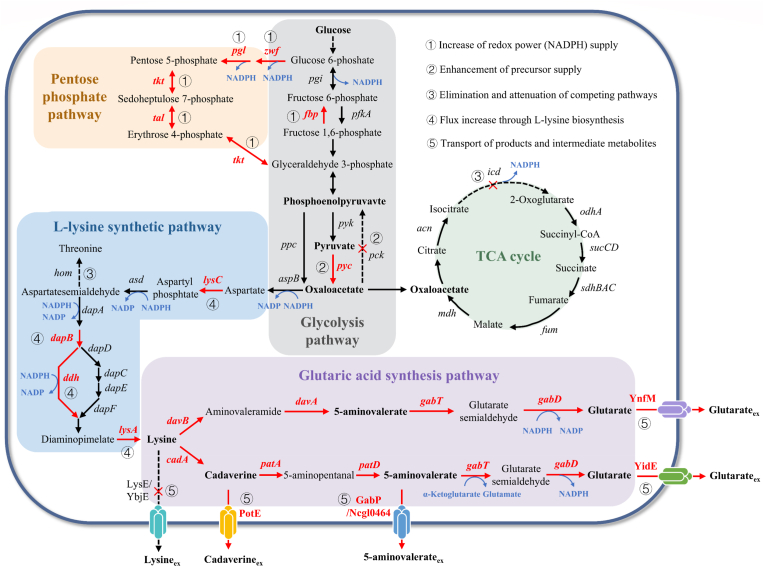
Design of glutaric acid production pathway based on system metabolic engineering. Here are the genes mentioned in the figure and their corresponding enzymes*: zwf*: glucose-6-phosphate 1-dehydrogenase; *pgl*: 6-phosphogluconolactonase; *tkt*: transketolase; *tal*: transaldolase; *pgi*: glucose-6-phosphate isomerase; *fbp*: fructose 1,6-bisphosphatase; *pfkA*: phosphofructokinase; *pyk*: pyruvate kinase; *ppc*: phosphoenolpyruvate carboxylase; *pyc*: pyruvate carboxylase; *pck*: phosphoenolpyruvate carboxykinase; *can*: aconitase; *icd*: isocitrate dehydrogenase; *odhA*: α-ketoglutarate dehydrogenase; *sucCD*: succinyl-CoA synthetase; *sdhBAC*: succinate dehydrogenase complex; *fum*: fumarase; *mdh*: malic dehydrogenase; *aspB*: aspartate aminotransferase; *lysC*: aspartokinase; *asd*: aspartate semialdehyde dehydrogenase; *hom*: homoserine dehydrogenase; *dapA*: dihydrodipicolinate synthase; *dapB*: dihydrodipicolinate reductase; *dapD*: tetrahydrodipicolinate succinylase; *dapC*: succinyl-amino-ketopimelate transaminase; *dapE*: succinyl-iaminopimelate desuccinylase; *dapF*: diaminopimelate epimerase; *ddh*: diainopimelate dehydrogenase; *lysA*: diaminopimelate decarboxylase; *davB*: lysine 2-monooxygenase; *cadA*: lysine decarboxylase; *davA*: 5-aminovaleramide amidohydrolase; *gabT*: 4-aminobutyrate-2-oxoglutarate transaminase; *gabD*: succinate-semialdehyde dehydrogenase; YnfM: glutarate export carrier in *C.glutamicum*; *patA*: putrescine aminotransferase; *patD*: aminobutyraldehyde dehydrogenase; YidE: glutarate export carrier in *E.coli*; LysE: l-lysine export carrier in *C. glutamicum*; YbjE: l-lysine export carrier in *E. coli*; PotE: cadaverine export carrier; Gabp: 5-aminovalerate export carrier in *E. coli*; Ncg10464: 5-aminovalerate export carrier in *C. glutamicum*.

Unlike conventional transformation strategies, system metabolic engineering employs a whole-cell, multi-module collaborative optimization approach. Integrating multi-omics analytic tools, including genomes, transcriptomics, and metabolomics, enables the precise identification of critical regulatory nodes and metabolic bottlenecks in the glutaric acid production pathway, facilitating the global optimization of metabolic networks (Fig. 2).

Li et al. developed a coordinated regulation strategy comprising multiple modules, grounded in the natural lysine catabolism mechanism of *E. coli* [41]. The strategy incorporates three primary components: (1) the enhancement of lysine biosynthesis through the supplementation of glutamate and NAD(P)H; (2) the alleviation of lysine feedback inhibition on critical enzymes; and (3) the optimization of oxaloacetate supply to maximize the directed carbon flux towards glutarate. The team successfully reduced the extracellular accumulation of intermediate metabolites, specifically cadaverine and 5-aminopentanoic acid, by identifying and overexpressing specific transporters. Under fed-batch fermentation conditions, the final engineered strain BM31PER achieved glutaric acid yield of 54.5 g/L and molar yield of 0.54 mol/mol glucose. This result verifies the high efficiency of carbon flux redirection and highlights the significant role of transporter engineering in minimizing metabolic intermediate leakage. In a recent study, Chu et al. constructed a plasmid-free recombinant *E. coli* based on system metabolic engineering to produce glutaric acid [42]. This study is distinguished from previous reports by its flexible application of biosensor and omics knowledge. Initially, the l-lysine producing strain LYS-14 was developed through ARTP(Atmospheric and Room Temperature Plasma) mutagenesis in conjunction with high-throughput screening utilizing lysine biosensors. Genome sequences of strain LYS-14 and its parent strain were compared to identify appropriate gene editing integration sites for the incorporation of the glutaric acid synthesis pathway into the chromosome of LYS-14. Subsequently, a glutarate biosensor was developed, whose sensing element consists of the regulatory protein CsiR in *P*. *putida* KT2440 and the PG promoter of the *csiD* gene. To enhance the rate-limiting DavTD module in the glutaric acid synthesis pathway, an optimized glutaric acid biosensor was employed to identify high-efficiency strains with an optimized gene copy number. Transcriptome analysis revealed a potential glutarate transporter, YidE, which was overexpressed in the genome, resulting in 38 %, 37.9 % and 7.1 % increases in glutarate titer, yield and productivity, respectively. In a 5 L fed-batch fermentation, the glutaric acid titer reached 44.8 g/L, with a yield of 0.28 g/g and a productivity of 0.62 g/L/h. The plasmid-free system developed in this study reduces resource competition between plasmid replication and host metabolism. This chromosomal integration of gene expression is more stable; at the same time, it eliminates the expression of antibiotic resistance markers, thereby reducing production costs. In addition, the high throughput screening strategy based on biosensors has shown application value in the breeding of glutaric acid producing strains. In another innovative study, Wang et al. systematically optimized the Cad pathway from l-lysine to glutaric acid using an in vitro pathway reconfiguration strategy [43]. The team first engineered ribosome binding sites (RBS) to fine-tune the molar ratio (4:8:7) of key enzymes *(E. coli* l-lysine decarboxylase EcCA, *Klebsiella pneumoniae* putrescine aminotransferase KpcPA, and gamma-aminopentanal dehydrogenase KpcPD) to balance metabolic flux. KpcPA was subsequently analyzed for volume and hydrophobicity through rational design, resulting in a significant enhancement of its catalytic activity for cadaverine. Overexpression of the gene *gabP*, which encodes the 5-aminopentanoic acid transporter, significantly decreased the extracellular accumulation of 5-aminopentanoic acid. The optimized strain Glu-02 achieved a glutaric acid titre of 77.62 g/L using 100 g/L l-lysine as substrate over a period of 42 h, resulting in a carbon molar conversion rate of 0.78 g/g, establishing a new record for glutaric acid production by *E. coli*.

Rohles et al. utilized *C. glutamicum* AVA-2, which produces 5-AVA, as the initial strain, overexpressing 5-aminopentanoate aminotransferase (GabT) and glutarate semialdehyde dehydrogenase (GabD) regulated by the constitutive promoter P*tuf*, thereby converting 5-AVA to glutarate [32]. To improve carbon flux utilization, the 5-AVA transporter NCgl0464 was overexpressed to reduce the accumulation of the intermediate metabolite 5-AVA. In a fed-batch process, engineered strain GTA-4 produced 90 g/L glutaric acid from glucose and molasses, with yields as high as 0.70 mol/mol glucose and maximum productivity of 1.8 g/L/h. Han et al. effectively engineered a microbial cell factory for the efficient manufacture of glutaric acid through systematic metabolic modifications of the l-lysine-producing *C. glutamicum* BE strain [29]. Firstly, l-lysine monooxygenase (*davB*) and 5-aminopentanamide hydrolase (*davA*) genes from *P*. *putida* and 4-aminobutyrate aminotransferase (*gabT*) and succinate semialdehyde dehydrogenase (*gabD*) genes from *C*. *glutamicum* were optimized to construct an efficient glutaric acid synthesis pathway. To enhance strain performance, genome-scale metabolic model analysis predicted critical metabolic nodes; comparative transcriptome analysis identified differentially expressed genes; and flux response analysis pinpointed metabolic bottlenecks. Through these investigations, the researchers meticulously controlled the expression levels of 11 target genes, thereby enhancing the availability of precursor l-lysine. The glutaric acid transporter encoded by the *ynfM* gene was first identified, and its genomic overexpression was driven by the H30 promoter. In fed-batch fermentation, the engineered strain GA17 (pGA4) harboring this overexpression cassette achieved glutaric acid production of 105.3 g/L, with a yield and productivity of 0.54 g/g and 1.53 g/L·h^−1^, respectively. These values were significantly higher than those of the parental strain BE (pGA4), which produced 54.4 g/L glutaric acid with a yield of 0.13 g/g and a productivity of 0.35 g/L·h^−1^. This study underscores the pivotal role of omics approaches in regulating intricate metabolic networks and reaffirms that product transport engineering is a crucial strategy in metabolic engineering.

In general, metabolic engineering can quickly and effectively obtain glutaric acid producing strains, but the improvement of later yield is easy to encounter bottlenecks; while system metabolic engineering can accurately break through bottlenecks and improve the production performance of glutaric acid engineering strains significantly. In the actual strain development work, the synergistic application of metabolic engineering and system metabolic engineering can improve the productivity of glutaric acid producing strains. It is noteworthy that transporter engineering can markedly enhance the production efficiency of glutaric acid in modified strains. The issues of low product efflux efficiency and loss of the intermediate metabolite are efficiently addressed by over-expressing the product transporter or knocking down the export protein of the intermediate metabolite. In addition, *C. glutamicum* demonstrates notable benefits in glutaric acid biosynthesis because to its superior l-lysine production capabilities and resilient commercial fermentation traits.

## 4 Future prospects and challenges

Despite advancements in metabolic engineering enhancing the efficiency of glutaric acid production in microbial cells, numerous critical scientific challenges and technical obstacles remain to be addressed. Innovative synthetic biology tools and systems biology methodologies offer novel solutions to these challenges. Moreover, the integration of artificial intelligence in enzyme design and pathway optimization, with high-throughput strain screening facilitated by microfluidic technology, is anticipated to yield significant advancements in the efficient biosynthesis of glutaric acid.

### 4.1 Reverse metabolic engineering based on omics analysis

**Fig. 3:**
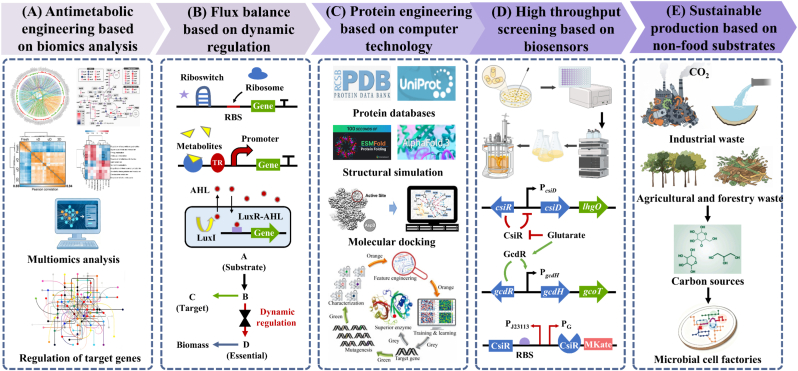
Prospect of green synthesis of glutaric acid.

Reverse metabolic engineering is the inverse approach to metabolic engineering, designed to enhance the production of target products by inhibiting or diminishing the accumulation of competitive routes, by-product creation pathways, or hazardous metabolites [44,45]. This strategy aims to minimize metabolic flux shunting through gene deletion, promoter modulation, or enzyme activity inhibition, thereby channeling increased carbon flow towards the target product [46,47]. The advancement of diverse omics technologies enables a thorough analysis of microbial metabolic regulation mechanisms, facilitating the precise identification of critical modification targets within metabolic pathways, thereby enhancing the synthesis efficiency of target products [[48], [49], [50]](Fig. 3A). The utilization of omics knowledge in the rational design of engineered strains offers significant theoretical insights for the metabolic network remodeling of microbial cell factories [51,52]. Subsequently, reverse metabolic engineering utilizing omics can amalgamate big data analytical technologies, including genomics, transcriptomics, proteomics, and metabolomics, to systematically identify and precisely manipulate competitive pathways, by-product synthesis, and toxic nodes within metabolic networks to enhance the biosynthesis of target products [[53], [54], [55]]. The identification of critical targets for enhancing glutaric acid production through multi-omics analysis and metabolic engineering led to the generation of 105.3 g/L of glutaric acid in 69 h by engineered strains, demonstrating the practical applicability of this strategy [29]. Nonetheless, the metabolic regulatory targets in this study have been recognized for an extended period as crucial genes for enhancing l-lysine production; thus, the metabolic regulatory mechanisms governing the glutaric acid synthesis pathway remain inadequately explored. Despite the robust data support that omics technology offers to metabolic engineering, it continues to encounter numerous hurdles in practical implementation. The measurement methodologies, temporal scales, and dynamic ranges of various omics data varies, complicating direct correlation; static omics data may not correctly represent the actual dynamic metabolic state of cells [56]. Furthermore, sophisticated technologies including AI-driven data fusion, real-time metabolomics integrated with microfluidic chips and mass spectrometry imaging (MSI), as well as multi-scale metabolic models, have been utilized in medical diagnosis and biochemical analysis, anticipated to assist researchers in developing more precise and rational cell metabolic engineering frameworks [[57], [58], [59]].

### 4.2 Flux balance based on dynamic regulation

Dynamic regulation refers to the ability of cells to modify gene expression or metabolic pathway activity instantaneously in response to environmental cues or alterations in internal metabolic conditions, hence optimizing cellular function or product synthesis [60,61]. In contrast to static regulation (such as constitutive expression or fixed induction), dynamic regulation allows microorganisms to adaptively allocate carbon flux over time, balancing growth and product synthesis, thereby significantly enhancing target metabolite titer and cellular adaptability [[62], [63], [64]]. Dynamic regulatory methodologies have emerged as a compelling subject in metabolic engineering, and gene regulatory elements have been successfully developed [65,66] (Fig. 3B). Glutaric acid, a catabolic byproduct of l-lysine, has a complex biochemical process originating from glucose and encompassing the synthesis of various intermediary intermediates. The equilibrium of flux across modules at various cellular growth phases is essential for cellular proliferation and product creation. Furthermore, the principal enzyme involved in glutaric acid synthesis is sourced from *P. putida*, and the expression of this heterologous enzyme invariably consumes ribosomes, tRNA, ATP, and other resources, leading to a metabolic burden on the host cells [64]. Genetic circuits based on the quorum sensing effect enable the integration of gene expression with cellular physiological status, and have been applied to induce recombinant protein expression as well as balance the carbon flux between cell growth and product synthesis [60]. Thus, the regulation of metabolic flux redistribution relying on quorum sensing can serve as an effective strategy for optimizing glutaric acid biosynthesis in engineered strains [67]. Furthermore, biosensors are capable of detecting variations in input signals (i.e., the concentrations of substrates or metabolites) and subsequently triggering a corresponding response as a consequence of detection [68]. The metabolic regulation approach of microbial cells based on transcriptional regulators has been successfully employed to improve host cell growth and the production of target compounds [69]. To date, two transcriptional regulators derived from *P*. *putida* have been reported to respond to intracellular fluctuations in glutaric acid concentration, and these transcriptional regulatory systems exhibit considerable application potential for the dynamic regulation of glutaric acid biosynthesis [70,71]. Notably, the response sensitivity and dynamic regulatory range of native transcriptional regulatory systems are often unable to meet the demands of metabolic regulation, and thus the optimization of response thresholds and sensitivities constitutes an indispensable step. Nonetheless, research on the dynamic regulation of glutaric acid production has yet to be documented. In future endeavors, metabolic engineers must focus on the creation and implementation of dynamic regulation techniques, which are expected to alleviate obstacles in enhancing cellular glutaric acid synthesis.

### 4.3 Protein engineering based on computer technology

The biosynthesis of glutaric acid in both *E. coli* and *C. glutamicum* relies on the heterologous expression of essential enzymes for glutaric acid production derived from *P. putida*. Currently, the enhancement of important enzyme expression levels has been implemented to surmount the metabolic bottleneck by traditional methods, including codon optimization, the utilization of robust promoters, and the augmentation of copy number. To maximize the industrial application efficacy of engineering strain in glutaric acid production, it is essential to optimize the structure of important enzymes to augment their activity, hence increasing the conversion efficiency of l-lysine to glutaric acid. Protein engineering is a method that modifies protein molecules by rational design or guided evolution to enhance their function, stability, or introduce new traits [[72], [73], [74]]. The primary objective is to acquire proteins that fulfill the requirements of particular applications. In recent years, the swift advancement of computing technology and artificial intelligence is propelling protein engineering into a closed-loop paradigm of “design-build-test-learn” (DBTL), offering robust tools for synthetic biology and precision medicine [75,76] (Fig. 3C). Despite the ongoing challenges of computational accuracy and the complexities of dynamic processes like allosteric effects, computer-aided protein engineering can substantially diminish trial and error expenses and expedite design cycles [77,78]. Molecular docking can be directly performed on enzymes with well-defined protein structures, while protein conformational changes can be examined through molecular dynamics (MD) simulations. A crucial enzyme mutant library can be generated by integrating subsequent site-directed mutagenesis, error-prone PCR, and other methodologies [[79], [80], [81]]. For proteins with unreported structures, such as DavA and DavB, essential enzymes in glutaric acid synthesis, researchers typically must examine the protein structure using X-ray crystallography and cryo-electron microscopy prior to undertaking further modification processes [82,83]. Currently, advanced big data intelligent computing technologies can directly forecast the structures of template-free proteins and perform homology modeling, exemplified by AlphaFold 2 and ESMFold, while generative models such as RFdiffusion and Chroma are capable of designing protein scaffolds [[84], [85], [86]]. Moreover, data-driven approaches and AI integration can aid researchers in modeling sequence-function links by employing deep learning techniques, such as Transformer models, to extract design principles from extensive datasets [87]. Computer-assisted directed evolution can reach virtual saturation mutations by simulating all conceivable single-point mutations and screening for potentially advantageous mutations [88,89].

### 4.4 High throughput screening based on biosensors

Biosensors can transform the concentration of certain metabolites into readily observable signals, including fluorescence, colorimetric changes, and growth rate variations [[90], [91], [92], [93]]. In recent years, biosensor-driven high-throughput screening has enabled the swift and effective identification of target phenotypes from extensive mutant libraries [94,95]. Transcription factors that react to metabolite target concentrations, subsequently activating transcription factor-regulated promoters to generate signal output proteins, represent the most traditional biosensors to date [69,96]. The glutarate biosensor follows the framework described above and consists of the glutarate-responsive transcription factor CsiR, the PG promoter driven by CsiR, and the red fluorescent protein mKate [42](Fig. 3D). The LysR family protein GcdR in *P. putida* regulates glutarate catabolism by promoting the transcription of two essential genes, *gcdH* and *gcoT*, in the glutaryl coenzyme A dehydrogenation pathway [70,71]. Consequently, the capability of the transcription factor GcdR to develop glutarate biosensors merits investigation. The present threshold of glutaric acid biosensors is 18 mM, significantly restricting the measurement of product titers in practical applications. Gradient strength promoters can typically be acquired via promoter engineering screening to modulate RNA polymerase binding efficiency, or the linear response range of biosensors can be expanded by modifying the quantity, location, and affinity of transcription factor binding sites to enhance the operator sequence [97,98]. In high-throughput screening technology, in addition to the traditional 96-microplate reading approach, modern techniques such as flow cytometry and microfluidic chips can be integrated to facilitate the sorting of cell fluorescence signals [[99], [100], [101]].

### 4.5 Sustainable production based on non-food substrates

Non-grain fermentation denotes the synthesis of target chemicals through microbial fermentation utilizing non-edible biomass (including CO_2_, industrial waste, lignocellulose, kitchen refuse, etc.) as a carbon source [5,15,16,102]. In contrast to conventional grain-based fermentation utilizing glucose, non-grain fermentation offers benefits such as circumventing rivalry for grain resources, lowering raw material expenses, and repurposing waste materials [[103], [104], [105], [106]]. Recently, the substrate range of *C. glutamicum* has been expanded through the heterologous expression of degradation and uptake proteins, leading to the enhanced production of l-lysine from diverse non-food substrates, including arabinose, xylose, glycerol, cellobiose, and carboxymethylcellulose [[107], [108], [109], [110]]. Nonetheless, prior experience indicates that modified strains, which broaden the substrate spectrum through heterologous expression of degrading enzymes, struggle to attain l-lysine synthesis at industrially applicable levels [108]. To enhance the sustainability of industrial production, techniques can be employed to alter engineered strains for the fermentation of glutaric acid using inexpensive and non-competitive carbon sources (Fig. 3E). Lignocellulose is the most abundant biomass of all and is considered a potential candidate to replace fossil resources for the synthesis of chemicals, materials, and fuels [16]. In order to achieve environmentally friendly and sustainable synthesis of glutaric acid, adaptive evolution or metabolic engineering can be used to improve the conversion rate of lignocellulose treatment solution. Lignocellulose is composed of cellulose, hemicellulose and lignin [15]. Cellulose can be hydrolyzed to glucose, hemicellulose can be hydrolyzed to xylose, arabinose, etc., while lignin can produce phenolic substances in the degradation process. Therefore, the costs associated with substrate collection, handling, and product isolation and purification need to be considered.

The swift advancement of AI-driven data integration and predictive modeling tools has revitalized synthetic biology approaches, including protein engineering, high-throughput screening, dynamic regulation, and omics analysis. Currently, metabolic engineering has transitioned from a “trial and error” approach to a “data-driven” precision design methodology. In the future, the integration of developing techniques such as artificial intelligence, synthetic biology, and single-cell technology will enable metabolic engineering to achieve more efficient and cost-effective biological production, hence offering essential support for green chemistry and sustainable development.

## 5 Conclusions

Glutaric acid, a significant C5 platform product, possesses extensive application potential in bio-based materials, medicine, and chemical engineering. This study emphasizes the substantial advancements in the construction and optimization of glutaric acid biosynthesis pathways by metabolic engineering. In the future, the integration of artificial intelligence in enzyme design, the advancement of efficient biosensors, the optimization of strains through dynamic regulation and omics techniques, and the utilization of non-grain substrates in fermentation are anticipated to facilitate the biosynthesis of glutaric acid, resulting in more efficient and cost-effective green manufacturing. The sustainable production methods of glutaric acid were reviewed, and some suggestions on its biosynthesis were put forward.

## CRediT authorship contribution statement

**Jie Liu:** Writing – original draft, Investigation, Funding acquisition, Formal analysis, Conceptualization. **Dan Mei:** Visualization, Investigation, Formal analysis. **Xuan-Jun Zhang:** Writing – review & editing, Funding acquisition. **Wei-Guo Zhang:** Writing – review & editing. **Long-Bao Zhu:** Writing – review & editing, Supervision.

## Declaration of competing interest

The authors declare that they have no known competing financial interests or personal relationships that could have appeared to influence the work reported in this paper.
